# Nanobody Mediated Inhibition of Attachment of F18 Fimbriae Expressing *Escherichia coli*

**DOI:** 10.1371/journal.pone.0114691

**Published:** 2014-12-11

**Authors:** Kristof Moonens, Maia De Kerpel, Annelies Coddens, Eric Cox, Els Pardon, Han Remaut, Henri De Greve

**Affiliations:** 1 Structural & Molecular Microbiology, Structural Biology Research Center, VIB, Brussels, Belgium; 2 Structural Biology Brussels, Vrije Universiteit Brussel, Pleinlaan 2, 1050 Brussels, Belgium; 3 Department of Veterinary Immunology, Faculty of Veterinary Medicine, Ghent University, Salisburylaan 133, Merelbeke 9820, Belgium; Centre National de la Recherche Scientifique, Aix-Marseille Université, France

## Abstract

Post-weaning diarrhea and edema disease caused by F18 fimbriated *E. coli* are important diseases in newly weaned piglets and lead to severe production losses in farming industry. Protective treatments against these infections have thus far limited efficacy. In this study we generated nanobodies directed against the lectin domain of the F18 fimbrial adhesin FedF and showed in an *in vitro* adherence assay that four unique nanobodies inhibit the attachment of F18 fimbriated *E. coli* bacteria to piglet enterocytes. Crystallization of the FedF lectin domain with the most potent inhibitory nanobodies revealed their mechanism of action. These either competed with the binding of the blood group antigen receptor on the FedF surface or induced a conformational change in which the CDR3 region of the nanobody displaces the D″-E loop adjacent to the binding site. This D″-E loop was previously shown to be required for the interaction between F18 fimbriated bacteria and blood group antigen receptors in a membrane context. This work demonstrates the feasibility of inhibiting the attachment of fimbriated pathogens by employing nanobodies directed against the adhesin domain.

## Introduction

In farming industry enterotoxigenic *Escherichia coli* (ETEC) and Shiga toxin producing *E. coli* (STEC) are important pathogens [1], [2] causing serious mortality and severe production losses [3]. Common to both classes of pathogenic *E. coli* is the presence of two crucial virulence factors: (1) adherence factors (often fimbriae) in order to mediate the attachment to specific receptors, usually glycans, followed by colonization of the intestinal tract and (2) the production of one or multiple toxins that induce disease symptoms [2]. In piglets ETEC and STEC strains expressing F18 fimbriae are associated with respectively post-weaning diarrhoea and edema disease [4], [5]. After the initial adherence step via the F18 fimbriae ETEC strains produce and secrete the heat-labile (LT) and/or heat-stable enterotoxins (ST), thereby stimulating the secretion of electrolytes and water and resulting in dehydration of the enterocytes and watery diarrhoea [6], [7]. F18 positive STEC strains instead produce the Shiga toxin Stx2e, which acts by depurination of a specific adenine from the 28S ribosomal RNA, efficiently shutting down protein synthesis and killing the affected cells that express the globotetraosylceramide receptor [8]. Damage to the vascular endothelium eventually results in edema, hemorrhage and microthrombosis, and will be fatal in 90% of all STEC affected animals [9]. STEC lack a secretory mechanism for Stx and the release of Stx occurs through lambdoid phage-mediated lysis [10].

F18 fimbriae are assembled by a dedicated machinery, the chaperone/usher pathway, that is distributed among genera of the phyla *Proteobacteria*, *Cyanobacteria*, and *Deinococcus-Thermus*. Essential to the CU pathway are a periplasmic chaperone protein and an outer membrane pilus assembly platform, termed usher [11]. Fimbrial subunits or pilins are stabilized by the chaperone and complemented in the final quaternary structure by an N-terminal donor strand of the following subunit. The usher both acts as a building platform and anchors the fimbriae to the cell surface [12]. The two-domain tipsubunit often harbors the adhesive properties and thus determines the host tropism of the bacteria [13]. It features a typical two-domain organization comprising an N-terminal receptor-binding domain linked to a C-terminal pilin domain that forms the connection to the fimbrial shaft. F18 fimbriae are comprised of the major subunit FedA [14], the minor subunit FedE and the tip-adhesin FedF [15], which binds to glycosphingolipids having A/H blood group type 1 core antigens (H: Fucα2Galβ3GlcNAcβ3Galβ4Glc; A: GalNAcα3(Fucα2)Galβ3GlcNAcβ3Galβ4Glc) that are present on enterocytes of the small intestine [16].

Previously we elucidated the co-complex structure of the FedF lectin domain with the blood group A type 1 hexasaccharide [17]. The carbohydrate ligand is interacting in an extended conformation at a shallow binding site on the FedF surface via an extensive hydrogen bond network. In addition we identified a polybasic loop, adjacent to the blood group binding site, which was shown a prerequisite for enterocyte binding although not required for glycan recognition in solution. Upon interaction of FedF with blood group antigen carrying sphingolipids, two positively charged lysine residues on this so called D″-E loop, identified by site-directed mutagenesis, we predicted to come into close proximity to the membrane. Both were shown be required for the FedF–glycosphingolipid interaction in proximity of the lipid bilayer and are expected to provide selectivity towards membrane-associated A/H determinants whilst evading binding to soluble A/H antigens on glycoproteins present in mucosal secretions.

After birth, suckling piglets are protected from F18^+^ STEC by specific antibodies in the sow milk. Vaccination of the sow can enhance the protection and so far several maternal vaccines are available on the market [18]. At weaning this lactogenic immunity is lost and together with the stress associated with the weaning period the piglet will become highly vulnerable to infections by enteric pathogens. Previously, antibiotics were widely employed as prophylaxis treatment during piglet weaning, but the recent ban of such therapy in the European Union has urged the development of alternative treatments. These include the administration of zinc oxide, organic acids, probiotics, prebiotics, antibodies and vaccines [1], [18], [19], but none provide a satisfying protection against ETEC and STEC caused disease. In addition, F18 fimbriae have a limited capability in stimulating the immune response after oral vaccination [18], [20].

In this study we identified specific nanobodies that could block attachment mediated by F18 producing *E. coli*. The inhibitory mechanism of four unique nanobodies that completely abolished adherence was investigated and shown to differ amongst them. Either the binding was prevented by competing for the carbohydrate binding site or a conformational change was induced in the critical D″-E loop. In the near future these nanobodies will be expressed in plants and used as a feed supplement against the economically important ETEC and STEC infections.

## Materials and Methods

### Generation and selection of anti-FedF_15–165_ nanobodies

The complete protocol of nanobody generation and selection by panning has recently been described thoroughly [21]. In short, a llama was immunized six times with 330 µg of purified FedF_15–165_ (see below) over a period of 6 weeks. Lymphocytes from the anti-coagulated blood of the immunized llama were employed to prepare cDNA that served as a template to amplify the open reading frames coding for the variable domains of the heavy chain antibodies. The PCR fragments were subsequently ligated into the pHEN11 phage display vector referred as pHEN4C in Conrath *et al*. [22]. The pHEN11 contains the chloramphenicol acetyltransferase gene replacing the beta-lactamase gene present in pHEN4. After transformation into *E. coli* TG1 cells, FedF_15–165_-specific nanobodies were selected by phage display [23]. After selection, phages were eluted by incubating the FedF_15–165_-coated wells with with 100 mM triethylamine (pH 10) for 10 min. After two rounds of panning, 96 individual colonies were selected and grown in 2X TY medium supplemented with chloramphenicol (25 µg/ml) and induced with 1 mM isopropyl β-D-1-thiogalactopyranoside (IPTG, Thermo Scientific)) for expression of soluble periplasmic nanobodies. The periplasmic extract was next subjected to an ELISA to confirm the selected nanobodies are indeed recognizing the purified FedF_15–165_. After the selection rounds the nanobody genes were PCR amplified using primers Lumio6 (5′-GGGGACCACTTTGTACAAGAAA GCTGGGTATTAGTGATGGTGATGGTGGTGTGAGGAGACGGTGACCTGGGTCCCCTGGCC-3′) and Lumio7 (5′-GGGGACAAGTTTGTACAAAAAAGCAGGCTTAAGAAGGAGATATACCATG AAATACCTATTGCCTACGGCAGCCGCTGGATTGT-3′) to introduce a His-tag at the C-terminus of the nanobodies. The resulting PCR fragments were inserted in the pDONR221 Gateway entry vector (Invitrogen) using the BP clonase enzyme (Invitrogen). Subsequently, the resulting plasmids were recombined into the pDESTR4-R3 plasmid, together with the araC activator and the pBAD promoter (pGV5159) and the green fluorescent protein encoded by the *gfp* gene (pENT105), in a MultiGateway reaction using the LR plus clonase enzyme (Invitrogen). The *gfp* gene was amplified from a GFP positive strain [24]. After transformation in CaCl_2_-competent *E. coli* DH5α, colonies were selected on LB-agar plates containing ampicillin (100 µg/ml). Transformants were PCR screened with primers Aida9b (5′-GCGAAATTAATACGACTCACTATA-3′) and pET-rv (5′-GGTTATGCTAG TTATTGCTCAGCG-3′).

### Expression, isolation and purification of the different FedF binding nanobodies

An overnight preculture of *E. coli* WK6 cells harboring the pDESTR4-R3 vector with insert encoding for 6xHis-tagged nanobodies was used to inoculate lysogeny broth (LB) media [25] (1∶100) supplemented with 100 µg/ml ampicillin. Bacterial cells were grown at 310 K, induced at OD_600 nm_ of ∼0.8 with arabinose (0.2%), 100 µg/ml ampicillin was added and the cell cultures incubated overnight at 310 K while shaking. The cell pellet was harvested by centrifugation at 6238 rcf for 15 minutes at 277 K. Extraction of periplasmic proteins was performed by suspending 1 g of wet cell pellet in 4 ml of 20 mM Tris-HCl pH 8, 2 mM EDTA, 30% (w/v) sucrose buffer. The mixture was left on ice for 30 minutes, centrifuged at 17418 rcf for 20 minutes at 277 K, and the cell pellet resuspended in 20 mM Tris-HCl pH 8.0 (4 ml/g pellet). The mixture was incubated half an hour on ice and centrifuged at 17418 rcf for 20 minutes at 277 K. The supernatant ( =  periplasmic extract) was collected and kept at 277 K until further purification.

To purify the different nanobodies 1 M NaCl was added to the filtrated periplasmic extract and subsequently loaded on a pre-packed Ni-NTA column (GE Healthcare) equilibrated in 20 mM Tris-HCl pH 8, 1 M NaCl. The column was washed with Tris-HCl pH 8, 1 M NaCl until all unbound contaminants were removed and eventually bound nanobodies were eluted by applying a stepwise gradient to 1 M imidazole. Elution fractions were analysed on purity with SDS-PAGE and dialyzed into the appropriate buffer solution.

### *In vitro* villous adhesion assay

F18 seronegative pigs, weaned at the age of 3–4 weeks, were transported to the stables of the Faculty of Veterinary Medicine (UGent). Upon arrival, they were orally given colistin (promycine pulvis; 1000 iu/mg; doses 0.1 g/kg) for 3 successive days to prevent or clear an infection with F18-positive *E. coli* which might occur at weaning. Pigs were euthanized by an overdose of Nembutal (60 mg/kg intravenously). The villi were prepared as previously described [26]. Briefly, a 20 cm long intestinal segment was excised of the mid jejunum at the moment of slaughter and washed twice with Krebs–Henseleit buffer (0.12 M NaCl, 0.014 M KCl, 0.001 M KH2PO4, 0.025 M NaHCO3, pH 7.4) and once with the same buffer containing 1% (v/v) formaldehyde for 30 min at 4°C. Subsequently, the villi were gently scraped from the mucosa with a glass slide and stored in Krebs–Heinseleit buffer at 4°C. When used for testing, villi were resuspended in PBS supplemented with 1% (w/v) D-mannose (Fluka) to prevent adhesion by type 1 pili.

An *in vitro* adhesion assay on small intestinal villous enterocytes was performed as previously described [27]. The wild type F18-positive *E. coli* strain 107/86 was mixed together with either 1× phosphate-buffered saline (PBS) buffer or one of the twelve different FedF-specific nanobodies (final concentration of 10 µg/ml) and tested for binding to villi by adding 4×10^8^ bacteria to an average of 50 villi in a total volume of 500 µl of PBS, followed by incubation at room temperature for 1 h while being gently shaken. Villi were examined by phase-contrast microscopy at a magnification of 600× and the number of bacteria adhering along 50 µm brush border was quantitatively evaluated by counting the number of adhering bacteria at 20 randomly selected places, after which the mean bacterial adhesion was calculated. Adhesion tests were performed in triplicate on intestinal villi of two different piglets.

### Expression and purification of FedF_15–165_

The N-terminal lectin domain of FedF comprising residues 15 to 165 (FedF_15–165_) was over expressed and purified as earlier described [28]. In short, plasmid pEXP62 was introduced in C43 (DE3) *E. coli* cells and grown in lysogeny broth (LB) supplemented with 100 mg/ml ampicillin at 37°C until the OD600 reached 1.0 and then induced with 1 mM IPTG. Induction was allowed to proceed during 3 h at 37°C, after which periplasmic proteins were collected by administering an osmotic shock. To purify FedF_15–165_ the cleared periplasmic extract was loaded onto a Source 30S cation exchange column equilibrated in 20 mM Tris-HCl pH 7.5 and eluted by a NaCl gradient to 0.5 M. Elution fractions were analyzed on purity with SDS-PAGE and dialyzed into the appropriate buffer solution.

### Microscale thermophoresis (MST)

Microscale thermophoresis is an immobilization-free technique for the analysis of biomolecules interacting in solution [29]–[31]. Saturation binding experiments were performed using a NanoTemper Monolith NT.115 instrument (NanoTemper Technologies). In MST an infrared-laser produces precise microscale temperature gradients within thin glass capillaries that are filled with the two binding partners. Molecules move along these temperature gradients and any change of the hydration shell of proteins due to changes in their primary, secondary, tertiary and/or quaternary structure affects the thermophoretic movement. FedF_15–165_ was fluorescently labeled via surface exposed amine groups with NT647 and the concentration was kept constant (150 nM) while varying the concentration of nanobodies, ranging from 1.28 µM to 0.04 nM. Afterwards 3–5 µl of the samples were loaded into glass capillaries (Monolith NT TM Capillaries) and the thermophoresis analysis was performed (LED 100%, IR laser 50%). The interaction was measured in HBS buffer (20 mM HEPES pH 7.4, 150 mM NaCl, 0.005% Tween20) and data was analyzed using the NT Analysis software (NanoTemper Technologies).

### Formation, crystallization and structure determination of the complexes between FedF_15–165_ and different inhibitory nanobodies

Complexes between FedF_15–165_ and the different nanobodies were obtained by adding an excess of FedF_15–165_ to the appropriate nanobody and subsequently load the mixture on a pre-packed Ni-NTA column (GE Healthcare). The FedF_15–165_-nanobody complexes will be retained on the column as only the nanobodies possess a C-terminal 6× His-tag. Elution of the complexes was performed by stepwise addition of 1 M imidazole.

The FedF_15–165_-NbFedF6, FedF_15–165_-NbFedF7 and FedF_15–165_-NbFedF9 co-complexes were dialyzed to 20 mM HEPES pH 7.5, 50 mM NaCl; concentrated and set up for crystallization (1∶1 stoichiometry protein/well solution). Initial hits were further optimized to conditions that produced well-diffracting crystals. FedF_15–165_-NbFedF6 (16 mg/ml) readily crystallized against a solution containing 160 mM NaCl, 80 mM Bis-Tris pH 5.5 and 20% PEG-3350; FedF_15–165_-NbFedF7 (7 mg/ml) against a solution 1 M (NH_4_)_2_ SO_4_, 90 mM Na-citrate pH 4.0; and finally FedF_15–165_-NbFedF9 (10 mg/ml) produced good diffracting crystals when set up against 2 M (NH_4_)_2_ SO_4_, 5% PEG-400 and 100 mM MES pH 6.5. The crystals were flash-cooled to 100 K in their crystallization solution supplemented with 15% glycerol for data collection. A single wavelength data was collected and data were processed with XDS and Xscale of XDS [32] and prepared with Pointless and Scala from the CCP4 suite [33]. Data were phased by molecular replacement [33] using coordinates of the previously determined FedF_15–165_ structure (PDB code 4B4P) and a random nanobody obtained from the PDB database (PDB code 2X1O). The models were further improved using the graphics program COOT [34] and refined using Refmac with TLS refinement [33] against the native dataset. Crystal parameters and data processing statistics for all structures are summarized in Table 1. The electron density maps of the interaction interface are shown in S5 Figure.

**Table 1:** Data collection, crystal parameters, and refinement statistics for the co-complexes of FedF_15-165_ with NbFedF9, NbFedF6 and NbFedF7.

	FedF_15-165_-NbFedF9	FedF_15-165_-NbFedF6	FedF_15-165_-NbFedF7
Wavelength	1.00	0.98	0.98
Beamline	SLS PX III	Diamond IO4	Soleil Proxima 2
Space group	C 2 2 2_1_	P 3_2_ 2 1	P 2_1_ 2_1_ 2_1_
a, b, c (Å)	52.6, 103.0, 114.5	101.4, 101.4, 62.8	30.3, 78.6, 108.9
α, β, g (°)	90, 90, 90	90, 90, 120	90, 90, 90
Resolution (Å)	46.95-1.57 (1.66-1.57)	87.8-2.51 (2.64-2.51)	44.77-1.88 (1.98-1.88)
No. of unique reflections	42810 (5989)	13039 (1868)	21548 (2703)
CC(1/2)	99.8 (56.1)	99.5 (79.3)	99.8 (74.3)
Rmeas (%)	14.4 (183.7)	18.7 (102.9)	11.2 (95.1)
Average I/σI	11.1 (1.0)	12.6 (2.7)	11.2 (2.1)
Completeness (%)	98.6 (95.5)	99.9 (99.4)	97.4 (85.6)
Multiplicity	9.7 (9.5)	10.9 (10.8)	5.7 (5.3)
Wilson B-factor	15.3	22.5	25.3
R_work_/R_free_(%)	19.5/22.6	20.0/25.4	18.6/22.6
average B-factor (Å^2^)	14.9	15.6	21.8
R.m.s. deviations			
Bond lengths (Å)	0.020	0.016	0.019
Bond angles (°)	1.968	1.869	1.994
No. Atoms (except H)			
Protein	2213	2001	2049
Water	228	57	88
Residues in allowed regions	99.1	98.8	99.2
(%) of Ramachandran plot			
PDB entry	4W6Y	4W6W	4W6X

a R*meas*  =  Σh (*n*h/*n*h-1) Σl |*Ihl* - <*Ih*>|/Σh Σl <*Ih*>, where *n*h  =  the number of observations for reflection **h**,Ihl  =  the intensity for observation **l** of reflection **h**, and <Ih>  =  the average intensity for reflection **h**.

b Statistics for outer resolution shell are given in parenthesis.

c Rwork  =  Σhkl ||Fobs | - |Fcalc ||/Σhkl |Fobs |.

d Rfree is defined as above but calculated for 5% of randomly chosen reflections that were excluded from the refinement.

### Surface plasmon resonance measurements

SPR experiments were carried out using a Biacore T200 instrument (GE healthcare). The surface of a CM5 sensor chip was activated with a 1∶1 mixture of 0.1 M N-hydroxysuccinimide (NHS) and 0.4 M 1-ethyl-3-(3-dimethylaminopropyl) carbodiimide hydrochloride (EDC). After activation of the surface the glycoconjugate human serum albumin-blood group A type 1 hexaose (HSA-A6-1) (10 µg/ml) (IsoSep AB, Sweden) in 10 mM sodium acetate, pH 4 was injected to flow cell 2 (FC2) to be immobilized on the sensor surface via primary amine groups. As a control the same amount of HSA was immobilized on FC1. Residual unreacted active ester groups were blocked with 1 M ethanolamine-HCl, pH 8.5. A constant amount of FedF_15–165_ (25 µM) was either alone or mixed with two-fold excess of the different nanobodies (50 µM) in HBS buffer (10 mM HEPES, 150 mM NaCl, 1 mM EDTA, 0.005% Tween20, pH 7.4) injected over the chip surface at a flow rate of 10 µl/min at 25°C.

### Ethics statement

Animal vaccination and experimentation was performed in strict accordance with good animal practices, following the EU animal welfare legislation and after approval of the local ethical committees [Committee for the Use of Laboratory Animals at the Vrije Universiteit Brussel and the Committee of the Faculty of Veterinary Medicine at the Universiteit Gent (EC 2014/02)]. Every effort was made to minimize animal suffering.

### Accession numbers

Atomic coordinates and structure factors have been deposited in the Protein Data Bank as identifiers 4W6W, 4W6X and 4W6Y.

## Results

### Nanobodies efficiently inhibit attachment of F18 fimbriated bacteria to piglet enterocytes *in vitro*

**Figure 1:**
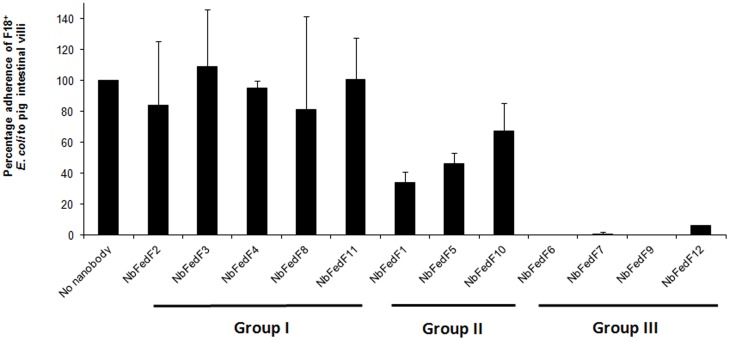
Blocking the attachment of F18 positive *E. coli* to piglet enterocytes by nanobodies. Nanobodies directed against the N-terminal domain of the FedF tipadhesin (FedF_15–165_) were assayed in an *in vitro* adherence test of wild type F18-positive *E. coli* strain 107/86 to piglet intestinal enterocytes. As a negative control PBS buffer was added instead of nanobody. Bacterial cells adhering to villi were counted under a microscope and plotted as a percentage of wild type binding.

**Figure 2:**
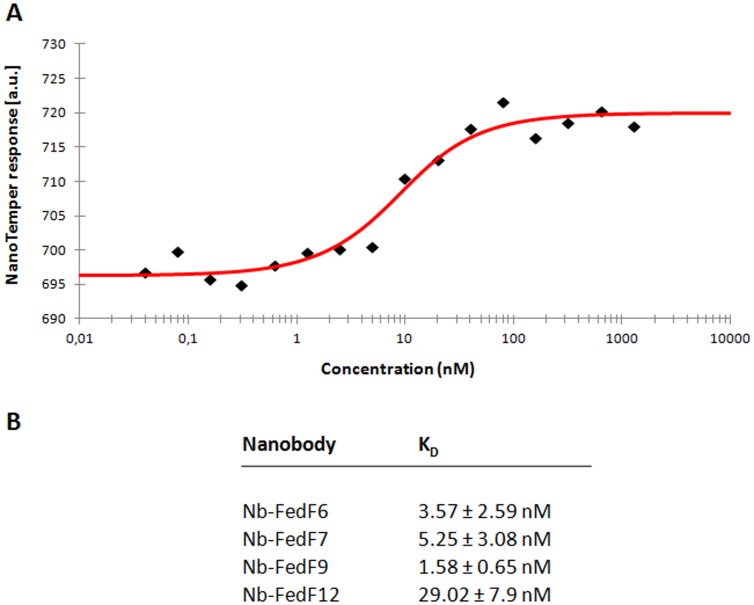
Inhibitory nanobodies recognize FedF_15–165_ with low nanomolar affinity. Microscale thermophoresis (MST) was employed to determine the in solution affinity between Nb-FedF6, Nb-FedF7, Nb-FedF9 and Nb-FedF12 with FedF_15–165_. (**A**) Typical MST measurement showing the interaction between Nb-FedF6 and FedF_15–165_. Data points are indicated by black diamonds, the fit by the NT Analysis software is shown as a red line. (**B**) Overview on the determined dissociation constants (K_D_) for the indicated Nb-FedF_15–165_ interactions.

A purified truncate (residues 15–165) of the FedF tipadhesin (FedF_15–165_) corresponding to the N-terminal lectin domain, where the binding capability to blood group type 1 antigens was shown to reside [17], was used to generate specific nanobodies [35]. Despite the smaller size of nanobodies compared to conventional antibodies they confer high affinity and antigen specificity, high stability and solubility, and are easy and inexpensive to produce [36]. A llama was immunized with FedF_15–165_ and specific nanobodies were selected by panning the immune library, derived from the llama peripheral blood lymphocytes, in two consecutive rounds using the phage display technology [21]. In total twelve nanobodies specific for FedF_15–165_ were obtained and further used to screen for their inhibitory capacity in an *in vitro* adherence assay on piglet enterocytes. The wild type *E. coli* strain 107/86 expressing F18 fimbriae [14] was incubated with each of the twelve different nanobodies and then added to the villi of piglets. The amount of bacteria adhering to the villi lining were counted, the results are summarized in Fig. 1 and showed that three categories of nanobodies could be distinguished. The adherence of the first group of nanobodies (NbFedF2, NbFedF3, NbFedF4, NbFedF8 and NbFedF11) remains unchanged compared to the control sample without added nanobody. In a second category, binding was reduced compared to wild type binding but still residual binding to the piglets enterocytes remained (NbFedF1, NbFedF5 and NbFedF10). A third group of nanobodies leads to the (near) complete loss of attachment of the wild type strain 107/86 to piglet villi (NbFedF6, NbFedF7, NbFedF9 and NbFedF12). Sequence alignment of these four inhibitory nanobodies reveals great sequence variability between them in all three complementary determining regions (S1 Figure), hinting that varying epitopes are recognized by these nanobodies. Microscale thermophoresis was used to determine the in solution affinity between FedF_15–165_ and the four inhibitory nanobodies (Fig. 2). Nanobodies NbFedF6, NbFedF7, NbFedF9 and NbFedF12 recognize FedF with low nanomolar affinity (Kd's of 3.57, 5.25, 1.58 and 29.02 nM, respectively), which is up to a thousand fold higher affinity in comparison with the interaction between FedF and the natural occurring glycan ligand blood group A type 1 hexasaccharide (2.9 µM) [17]. In the next sections we further characterized the three best inhibiting nanobodies (NbFedF6, NbFedF7 and NbFedF9) by X-ray crystallography.

### Nanobody NbFedF9 inhibits attachment by steric occlusion of the FedF carbohydrate binding site

**Figure 3:**
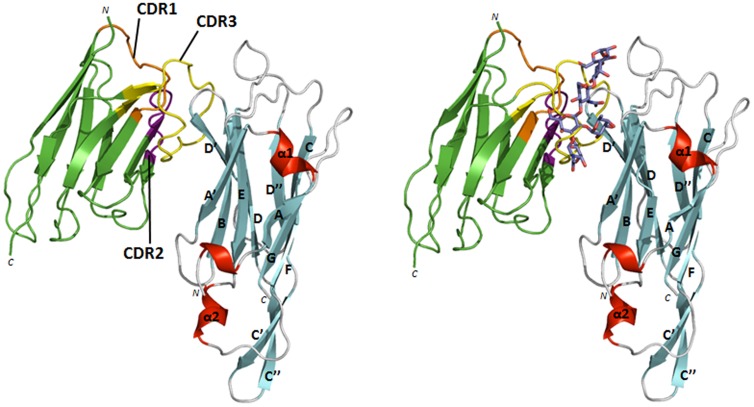
NbFedF9 inhibits the binding of F18 fimbriated *E. coli* to piglet enterocytes by occupying the carbohydrate binding site on the FedF surface. Left: structure of the complex between the inhibitory NbFedF9 (green) and FedF_15–165_ (β-strands, α-helices and loops are colored respectively cyan, red and grey), which shows NbFedF9 interacting at the side of the FedF fold. The three complementary determining regions (CDRs) of NbFedF9 are colored respectively in orange, purple and yellow. Right: overlay of the NbFedF9-FedF_15–165_ structure with the previously elucidated structure of the co-complex between FedF_15-165_ and the blood group A type 1 hexasaccharide [17]. Both the carbohydrate ligand and NbFedF9 compete for the same binding site on the FedF fold. Blood group A type 1 hexasaccharide is depicted in stick model with carbon, oxygen and nitrogen atoms colored respectively purple, red and blue.

Crystallization trials of the NbFedF9-FedF_15–165_ co-complex were set up in order to obtain structural information about the inhibitory mechanism of NbFedF9. These efforts resulted in the elucidation of the FedF_15–165_- NbFedF9 structure to a resolution of 1.5 angstrom using molecular replacement with the earlier determined apo-FedF_15–165_ structure (PDB identifier 4B4P). NbFedF9 interacts along the side of the FedF immunoglobulin-like fold (Fig. 3 left) by exclusively hydrogen bond formation, either directly by the interaction between residues of both NbFedF9 and FedF_15–165_ or indirectly with intermediary water molecules (S2 Figure). All but one of the interactions between NbFedF9 and FedF_15–165_ are governed by CDR3, which is more expanded in nanobodies compared to their VHVL antibody counterpart. Important direct interactions that stabilize the complex are hydrogen bonds between the side chains of His88 (FedF) and Tyr114 (NbFedF9), Arg117 (FedF) and Glu101 (NbFedF9), Glu122 (FedF) and Arg112 (NbFedF9), Glu96 (FedF) and Arg108 (NbFedF9) (S2 Figure). As well two direct interactions involving only main chain atoms are formed, more precisely between the amide group of Ile94 (FedF) and the carboxyl group of Arg108 (NbFedF9) and between the carboxyl group of Gly92 (FedF) and the amide group of Ser110 (NbFedF9) (S2 Figure). When the structure of the previously elucidated co-complex between FedF_15–165_ and the blood group A type 1 hexasaccharide (A6-1)[17] is overlaid on the NbFedF9-FedF_15–165_ complex it shows clearly how the binding sites for the glycan A6-1 and NbFedF9 on the surface of FedF are overlapping (Fig. 3 right; S3 Figure). Amino acid residues His88 and Arg117 on the FedF surface have been shown to be crucial in mediating the attachment of F18-fimbriated bacteria towards enterocytes in a mutational study [17]. In the FedF- NbFedF9 crystal structure these residues are involved in the formation of hydrogen bonds with NbFedF9 and thus unable to interact with the A6-1 ligand (Fig. 3; S3 Figure). All together the presented co-complex structure demonstrates how NbFedF9 inhibits the attachment of F18 fimbriated *E. coli* to villi by directly competing with the carbohydrate binding site on the surface of FedF.

### Nanobodies NbFedF6 and NbFedF7 inhibit attachment by inducing local conformational changes in FedF

**Figure 4:**
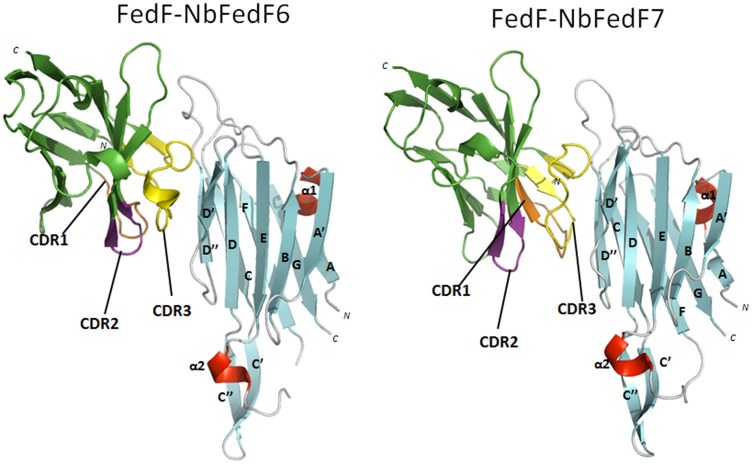
Overview of the binding of inhibitory nanobodies NbFedF6 and NbFedF7 on the surface of the F18 fimbrial adhesin FedF. Nanobodies NbFedF6 and NbFedF7 (green) are interacting with a near identical epitope at the interface of the two β-sheets that make up the immunoglobulin-like fold of FedF_15–165_ (β-strands, α-helices and loops are colored respectively cyan, red and grey). The three complementary determining regions (CDRs) are colored respectively in orange, purple and yellow.

**Figure 5:**
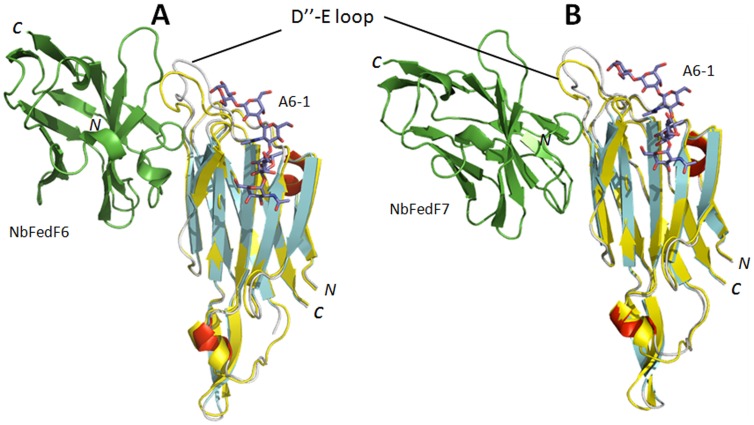
Conformational change induced inhibition of adhesion by nanobodies NbFedF6 and NbFedF7. Superimposition of the FedF-blood group A type 1 hexasaccharide (A6-1) co-complex structure (colored yellow) with the co-complex structures of FedF-NbFedF6 (**A**) and FedF-NbFedF7 (**B**) (β-strands, α-helices and loops are colored respectively cyan, red and grey). Both NbFedF6 and NbFedF7 induce a conformational change in the D″-E loop that is protruding from the FedF surface thereby reorienting the loop outwards from the A6-1 binding site. A6-1 is depicted in stick model with carbon, oxygen and nitrogen atoms colored respectively purple, red and blue.

**Figure 6:**
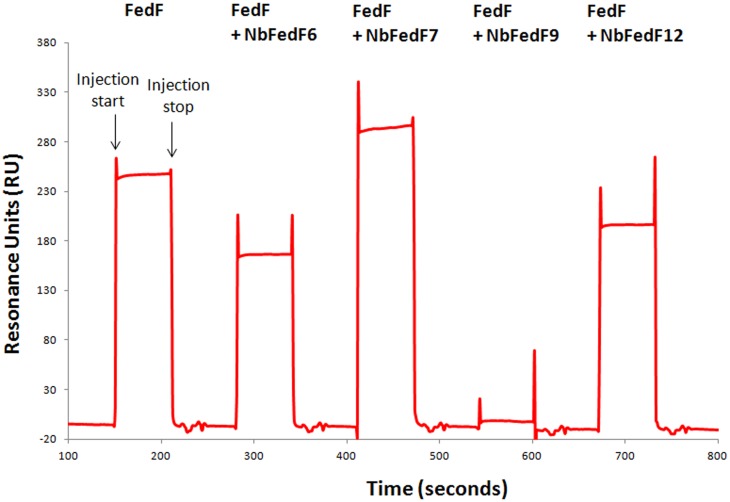
Nanobodies that induce a conformational change in the D″-E loop do not inhibit the attachment of FedF towards the A6-1 carbohydrate. Consecutive injections of either FedF_15–165_ or FedF_15–165_-nanobody complexes over the sensor chip surface carrying an immobilized A6-1-human serum albumin glycoconjugate were performed. Nanobody NbFedF9, shown in the crystal structure to bind in the FedF carbohydrate binding site, completely blocks the FedF-A6-1 interaction. In contrary, nanobodies NbFedF6, NbFedF7 and NbFedF12 only slightly or not at all inhibit the binding of FedF on the A6-1 coated surface. The crystal structures show how NbFedF6 and NbFedF7 induce a conformational change in the D″-E loop but do not steric compete with A6-1 binding.

Crystal structures of the complexes between FedF_15–165_ and both NbFedF6 and NbFedF7 were as well obtained to a resolution of respectively 2.5 Å and 1.7 Å (Fig. 4). In both elucidated complexes the nanobodies are occupying an overlapping epitope formed by strands D′ and D″ at the interface between the two β-sheets of FedF (Fig. 4). Nearly all interactions between NbFedF6 and FedF are mediated by the CDR3 loop. In the NbFedF7-FedF complex the contribution of CDR3 to the total binding affinity is even more pronounced as the other CDRs are not involved in any direct hydrogen bond formation at all. Direct hydrogen bond interactions that stabilize the NbFedF6-FedF_15–165_ complex are Arg45 (NbFedF6) and Ala96 (FedF), Ser54 (NbFedF6) and Gln84 (FedF), Phe100 (NbFedF6) and both Gly92/Asn81 (FedF), Tyr102 (NbFedF6) and Asn81 (FedF), Gln109 (NbFedF6) and both Gly98/Ala96 (FedF), Ala110 (NbFedF6) and Gly94 (FedF) (S4 Figure). In the NbFedF7-FedF complex Trp111 is inserted in a deep hydrophobic groove on the FedF surface with optimal shape complementary and as well forms a direct interaction with Ser79; other important interactions are Ser100 (NbFedF7) and Thr46 (FedF), Asn101 (NbFedF7) and both Asn81/Gly92 (FedF), Ser102 (NbFedF7) and Gln91 (FedF), Ala110 (NbFedF7) and Gly94 (FedF), Asn113 (NbFedF7) and Ser44 (FedF) (S4 Figure). Although a near identical epitope is targeted by NbFedF6 and NbFedF7 they differ significantly in the sequence of their CDR3 loop (S1 Figure) and the residues involved in the recognition of the epitope on the FedF surface. Both nanobody NbFedF6 and NbFedF7 are interacting distant from the A6-1 binding site (Fig. 5), thus contrary to the inhibitory complex between FedF- NbFedF9 in which NbFedF9 directly competed with the blood group antigen binding site. When superimposing the crystal structures of the FedF-A6-1 complex with the NbFedF6/7-FedF complex it shows how both nanobodies induce a conformational change in the D″-E loop (Fig. 5). The D″-E loop is displaced more outwards relative to the A6-1 binding site by the CDR3 loop of the nanobody and thereby pushed slightly upwards relative to the FedF surface. The conformation of none of the amino acid residues identified in our earlier study to interact with the A6-1 ligand is affected significantly [17]. To confirm that both nanobodies can still bind the ligand we performed an inhibition experiment using surface plasmon resonance. FedF was mixed with a fixed concentration of the different inhibitory nanobodies and injected over a chip on which a human serum albumine-A6-1 glycoconjugate was immobilized. As could be expected NbFedF9 completely abolished the binding of FedF with A6-1 (Fig 6). On the contrary when adding an excess of nanobodies NbFedF6 and NbFedF7, and as well nanobody NbFedF12, FedF was still able to fully or partially interact with the A6-1-HSA glycoconjugate (Fig. 6), and in addition these nanobodies are not altering the binding kinetics of the interaction. These results demonstrate the conformational change induced by the nanobodies NbFedF6 and NbFedF7 cannot completely explain their inhibitory capacity. This is despite their full binding inhibition in a biological context, when FedF is binding membrane-embedded sphingolipids. The D″-E loop harbors two positively charged lysine residues that we identified previously to add non-specific binding affinity in proximity to the membrane [17]. By targeting this loop and changing its conformation we could thus block the affinity of F18 fimbriated bacteria towards membrane imbedded glycosphingolipid receptors. Either this inhibitory effect stems from the disruption of the conformation of the critical D″-E loop, or another possibility that cannot be excluded is that NbFedF6 and NbFedF7 upon binding impart steric hindrance with the nearby phospholipid bilayer.

## Discussion

Adhesion of pathogens to receptors presented on the host tissue is the first crucial step in the initiation of infection. Often this goal is attained by hair-like surface organelles termed fimbriae or pili that enable the bacteria to reach distant located receptors without the immediate need for cell-cell contact. Enterotoxigenic *E. coli* and Shiga toxin producing *E. coli* strains causing respectively post-weaning diarrhoea and edema disease are important pathogenic strains and cause massive havoc in livestock. In order to infect piglets these strains express the F18 fimbriae on their cell surface that interacts via the two-domain FedF tipadhesin with ABO blood group type 1 determinants presented on glycosphingolipids. The molecular details on the FedF-blood group antigen interaction have been unraveled and directed mutagenesis together with *in vitro* binding assays demonstrated a polybasic D″-E loop adjacent to the carbohydrate binding site is required for to the FedF–glycosphingolipid interaction in proximity of the lipid bilayer [17]. The D″-E loop was proposed to direct selectivity towards membrane-associated blood group determinants and thus evades the binding to soluble glycoproteins that contain ABH blood group antigens, and are present in mucosal secretions. In this study we generated nanobodies that could block the attachment of F18 fimbriated *E. coli* cells to piglet villi in an *in vitro* cell binding assay. The co-complex crystal structures revealed the inhibitory action of these nanobodies, either they steric compete with the blood group antigen binding site on the FedF surface (NbFedF9) or they induced a conformational change in the crucial D″-E loop (NbFedF6, NbFedF7 and NbFedF12). This result highlights the importance of the critical D″-E loop in mediating attachment of F18 fimbriae with membrane localized receptors, although steric hindrance with the near membrane bilayer cannot be left out.

The FedF adhesin is highly conserved in isolates from different countries [37], and none of the amino acid variability within these isolates coincides with the epitope recognized by the different inhibiting nanobodies. Since these epitopes are highly conserved the nanobodies we described can be broadly used over different countries to neutralize the binding of F18 fimbriated ETEC and STEC strains on the villi of piglets and alleviate the symptoms caused by these infections. Oral administration of purified antibodies to the intestinal tract can be limited by the harsh acidic environment of the stomach [38]. In Virdi *et al.* (2013) we showed protection of weaned piglets by oral passive immunization with anti-F4 fimbriae nanobodies fused to the Fc domain of pig IgA by producing these fusion antibodies in *A. thaliana* seeds and administrating these crushed seeds as a feed supplement [39]. During transit through the gastrointestinal tract these fusion nanobodies are protected and can functionally realize their protective effect as evidenced by a progressive decline in shedding of F4-positive bacteria, a significantly lower immune response to F4 fimbriae and a higher weight gain compared to piglets in the non-treated control group [39]. It is anticipated that the anti-FedF nanobodies described in this paper can in a similar way be grafted to the Fc domain of pig IgA and produced in plant seeds to act as prophylactic agents. Both F4 and F18 positive strains have a high prevalence in post-weaning *E. coli* infections in piglets. The most effective protection against ETEC and STEC infections is therefore expected to come from passive immunization regimens that combine both anti-F4 and anti-FedF Fc fused nanobodies. F4 fimbriae have their adhesive capability build into the polymerizing subunit, resulting in the exposure of several hundreds of interaction sites across the fimbrial length. In contrast F18 fimbriae have a single tip-adhesin FedF that confers binding properties and thus less interaction surfaces need to be blocked to obtain a protective effect.

The usage of fusion nanobodies has a number of advantages over more conventional prophylactic treatments, like the use of vaccines [18] or small molecule compounds. Initially the generation of transgenic plants is labor intensive and costly, but afterwards production will be inexpensive and straightforward. Nanobodies feature a high affinity towards their respective antigens, whereas organic compounds often require several intensive rounds of structure-based chemical optimization to attain a reasonable binding affinity. Anti-adhesives designed against the FimH adhesin of type 1 pili, involved in the disease process of uropathogenic *E. coli*, show low nanomolar binding affinities [40]–[42] and were shown to be effective in an *in vivo* murine model upon oral administration [43]. FimH is however exceptional as it features a deeply buried mannose binding pocket that exhibits nanomolar binding strength towards its natural carbohydrate ligands [44]. Difficulties arise when targeting more open, shallow binding grooves as in the case for the PapG adhesin from P pili that are involved in adherence to the kidney epithelium, thereby causing pyelonephritis [45]. The most potent multivalent PapG inhibitor to date has an IC50 value of only 2 µM [46], [47]. The majority of hitherto obtained crystal structures, including FedF, fall into this last class that possess shallow carbohydrate binding grooves and the macroscopic binding affinity of bacteria stems from the combined avidity of individual weak interactions. Together these observations indicate the problems associated with the approach of designing anti-adhesive organic compounds [48]. Nanobodies circumvent the long tedious and repetitive process of developing optimized small molecule anti-adhesives and will in a short time span result in nanomolar binding tools that block the unwanted attachment of bacterial lectins to host tissue.

In conclusion, we have generated and characterized four nanobodies that interfere with F18 fimbriae mediated attachment. The inhibitory mechanism has been unraveled and demonstrates the nanobodies directly compete with the blood group antigen binding site or induce a conformational change in the polybasic D″-E loop. These nanobodies will in the near future be expressed in plant seeds to act as a prophylactic agent to reduce the burden of ETEC and STEC infections in intensive pig farming.

## Supporting Information

S1 Figure **Sequence alignment of the four nanobodies that inhibit attachment of F18 positive** ***E. coli*** **with piglet villi** ***in vitro.*** Residues are colored according to the sequence variability between the nanobody sequences, with residues colored blue being the highest conserved and residues colored red exhibit the least conservancy. The three complementary determining regions (CDR) are indicated by green bars and named. Alignment was generated using CLC workbench.
(TIF)

S2 Figure **Details on the interaction site of NbFedF9 and FedF_15–165_.** NbFedF9 (yellow) binds at the side of the FedF fold (grey) and interacts solely by the formation of hydrogen bonds. Either direct hydrogen bonds (dashed lines, colored red) are formed by residues of both NbFedF9 and FedF_15–165_ or indirectly by an intermediary water molecule (dashed lines, orange). Amino acid residues involved in the interaction are named and indicated by either a black (NbFedF9) or dark blue (FedF) label. Water molecules are depicted as spheres and colored green. Interacting main chain and side chain atoms are depicted in stick representation with oxygen and nitrogen atoms colored respectively in red and blue.
(TIF)

S3 Figure **NbFedF9 directly competes with the sugar binding site on the FedF surface.** Shown is a comparison of the binding site of blood group A type 1 hexasaccharide (A6-1) (left) and NbFedF9 (right) on the FedF surface. Residues His88 and Arg117 can be seen to interact both with NbFedF9 and A6-1, and these residues are named. FedF is depicted in cartoon representation and colored gray, whereas NbFedF9 and A6-1 are depicted in cartoon and stick representations, respectively, and colored yellow. Interacting residues are shown in stick model with oxygen and nitrogen atoms colored red and blue, respectively. Hydrogen bonds are highlighted as red dotted lines.
(TIF)

S4 Figure **Details on the interaction between FedF_15–165_ and NbFedF6 or NbFedF7 that induce a conformational change in the D″-E loop**_._ NbFedF6 (**A**)(**B**) and NbFedF7 (**C**)(**D**) are colored yellow and interact at the interface between the two β-sheets of the immunoglobulin-like fold of FedF (grey). Direct hydrogen bonds (dashed lines, colored red) are formed by residues of both nanobodies and FedF_15–165_ or indirect hydrogen bonds by a connecting intermediary water molecule (dashed lines, orange). Amino acid residues involved in the interaction are named and indicated by either a black (nanobodies) or dark blue (FedF) label. Water molecules are depicted as spheres and colored green. Interacting main chain and side chain atoms are depicted in stick representation with oxygen and nitrogen atoms colored respectively in red and blue.
(TIF)

S5 Figure **Electron density maps of the interface of the different FedF_15–165_-nanobody complexes.** Electron density map at 1.6 sigma of the interaction interface of the FedF_15–165_-NbFedF6, FedF_15–165_-NbFedF7 and FedF_15–165_-NbFedF9 complexes.
(TIF)
