# Supplementary figures and images for: Nanobody Mediated Inhibition of Attachment of F18 Fimbriae Expressing Escherichia coli
Source: PLoS One. 2014 Dec 11;9(12):e114691. doi: 10.1371/journal.pone.0114691 (PMC4263667; doi:10.1371/journal.pone.0114691)

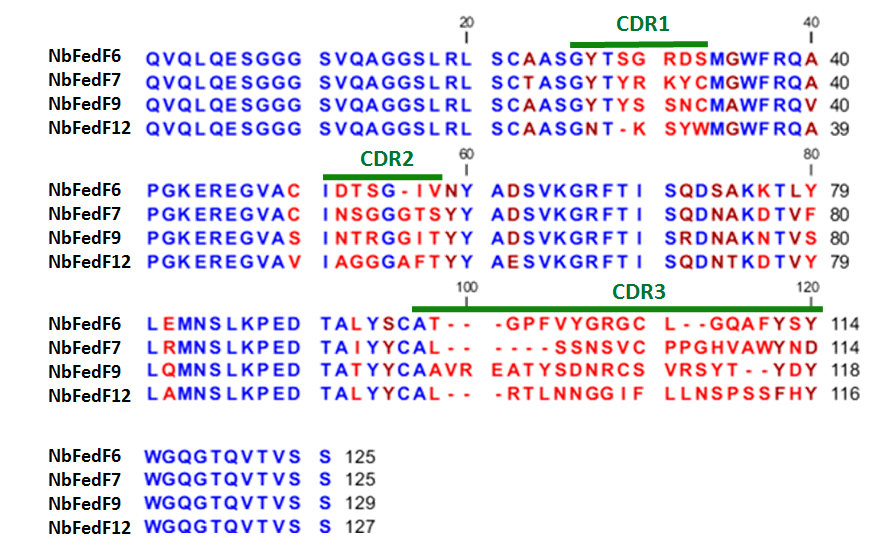

Supplement: S1 Figure — Sequence alignment of the four nanobodies that inhibit attachment of F18 positive E. coli with piglet villi in vitro. Residues are colored according to the sequence variability between the nanobody sequences, with residues colored blue being the highest conserved and residues colored red exhibit the least conservancy. The three complementary determining regions (CDR) are indicated by green bars and named. Alignment was generated using CLC workbench. (TIF) [file pone.0114691.s001.tif]

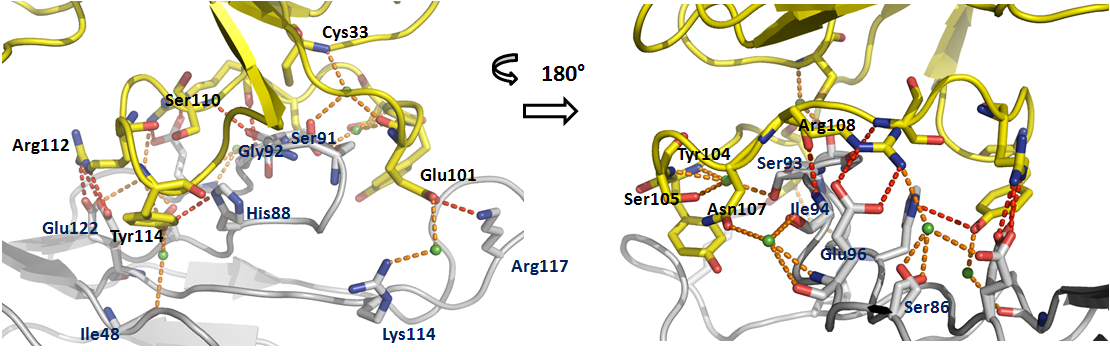

Supplement: S2 Figure — Details on the interaction site of NbFedF9 and FedF15–165. NbFedF9 (yellow) binds at the side of the FedF fold (grey) and interacts solely by the formation of hydrogen bonds. Either direct hydrogen bonds (dashed lines, colored red) are formed by residues of both NbFedF9 and FedF15–165 or indirectly by an intermediary water molecule (dashed lines, orange). Amino acid residues involved in the interaction are named and indicated by either a black (NbFedF9) or dark blue (FedF) label. Water molecules are depicted as spheres and colored green. Interacting main chain and side chain atoms are depicted in stick representation with oxygen and nitrogen atoms colored respectively in red and blue. (TIF) [file pone.0114691.s002.tif]

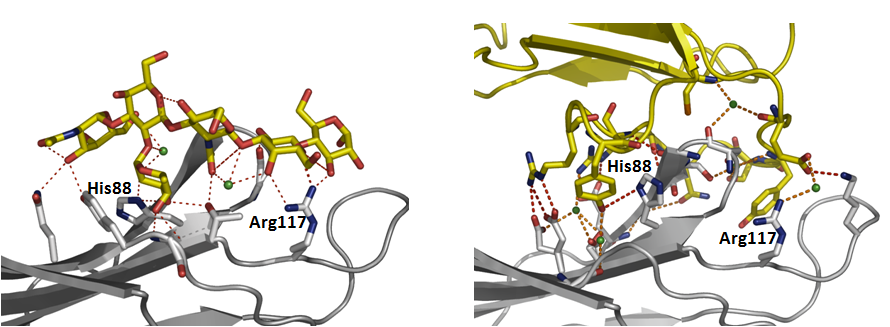

Supplement: S3 Figure — NbFedF9 directly competes with the sugar binding site on the FedF surface. Shown is a comparison of the binding site of blood group A type 1 hexasaccharide (A6-1) (left) and NbFedF9 (right) on the FedF surface. Residues His88 and Arg117 can be seen to interact both with NbFedF9 and A6-1, and these residues are named. FedF is depicted in cartoon representation and colored gray, whereas NbFedF9 and A6-1 are depicted in cartoon and stick representations, respectively, and colored yellow. Interacting residues are shown in stick model with oxygen and nitrogen atoms colored red and blue, respectively. Hydrogen bonds are highlighted as red dotted lines. (TIF) [file pone.0114691.s003.tif]

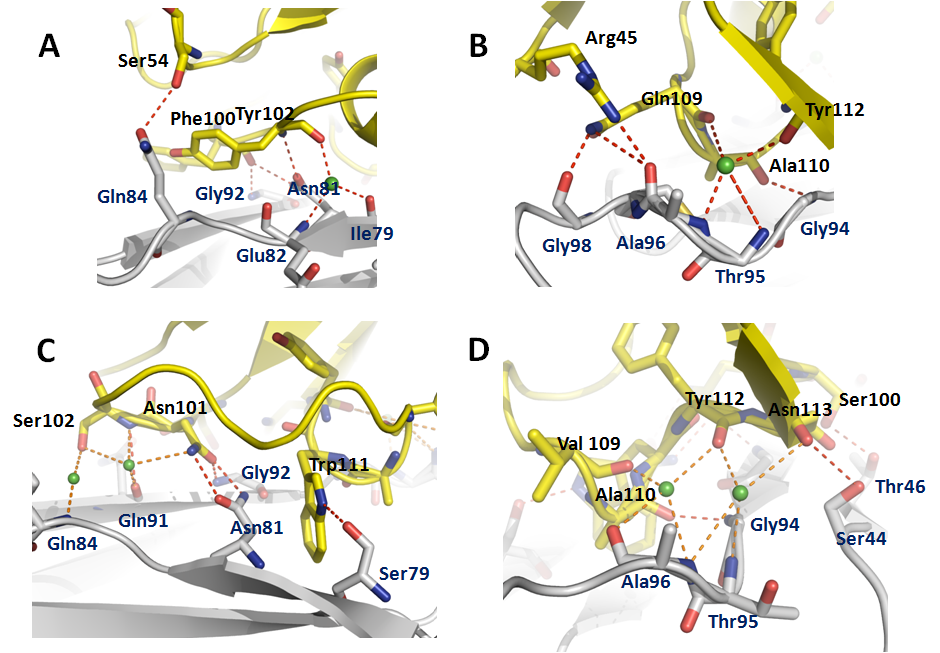

Supplement: S4 Figure — Details on the interaction between FedF15–165 and NbFedF6 or NbFedF7 that induce a conformational change in the D″-E loop . NbFedF6 (A)(B) and NbFedF7 (C)(D) are colored yellow and interact at the interface between the two β-sheets of the immunoglobulin-like fold of FedF (grey). Direct hydrogen bonds (dashed lines, colored red) are formed by residues of both nanobodies and FedF15–165 or indirect hydrogen bonds by a connecting intermediary water molecule (dashed lines, orange). Amino acid residues involved in the interaction are named and indicated by either a black (nanobodies) or dark blue (FedF) label. Water molecules are depicted as spheres and colored green. Interacting main chain and side chain atoms are depicted in stick representation with oxygen and nitrogen atoms colored respectively in red and blue. (TIF) [file pone.0114691.s004.tif]

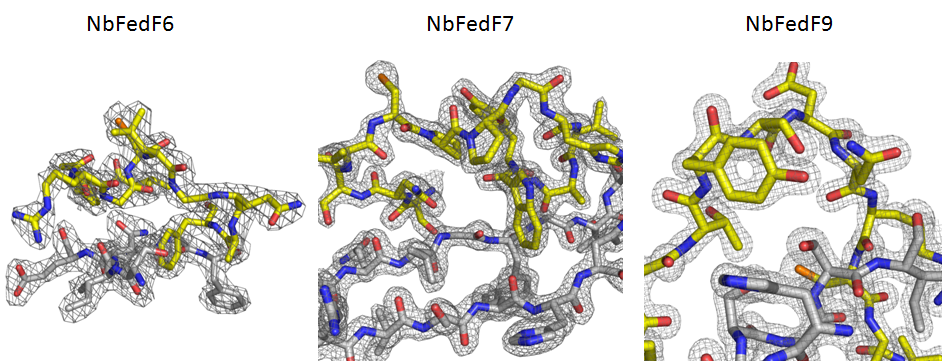

Supplement: S5 Figure — Electron density maps of the interface of the different FedF15–165-nanobody complexes. Electron density map at 1.6 sigma of the interaction interface of the FedF15–165-NbFedF6, FedF15–165-NbFedF7 and FedF15–165-NbFedF9 complexes. (TIF) [file pone.0114691.s005.tif]
